# Effects of Iron Deficiency on Cognitive Function in School Going Adolescent Females in Rural Area of Central India

**DOI:** 10.1155/2013/819136

**Published:** 2013-12-10

**Authors:** Sarika More, V. B. Shivkumar, Nitin Gangane, Sumeet Shende

**Affiliations:** ^1^Department of Pathology, Sri Lakshmi Narayana Institute of Medical Sciences, Osudu, Kudapakkam Post, Villanur Communie, Pondicherry 605502, India; ^2^Department of Pathology, Mahatma Gandhi Institute of Medical Sciences, Sevagram, Maharashtra, India; ^3^Department of Forensic Medicine, Sri Lakshmi Narayana Institute of Medical Sciences, Pondicherry 605502, India

## Abstract

Iron deficiency anemia is most common nutritional deficiency disorder in India and remains a formidable health challenge. Girls in the period of later school age and early adolescence are prone to develop iron deficiency. Iron deficiency leads to many non-hematological disturbances which include growth and development, depressed immune function in infants; reduces physical work capacity; decreases the cognitive function in both infants and adolescents. Present study was done to know the prevalence of iron deficiency in both the anemic and non anemic school going adolescent girls, to assess the effect of iron deficiency on cognitive functions in anemic iron deficient and non-anemic iron deficient school girls in a village school situated in central India. *Methods*. A secondary school having girl students in the age group of 12–15 years studying in sixth to ninth standard was selected. Serum ferritin concentration was estimated by ELISA. For assessing the cognitive function mathematics score, one multi-component test for memory, attention and verbal learning and Intelligent Quotient scores of the students were used. *Results*. Scholastic Performance, IQ and Scores of Mental balance, Attention & Concentration, Verbal Memory and Recognition were decreased in iron deficient girls, both anemic and non anemic as compared to the non iron deficient girls.

## 1. Introduction

Iron deficiency is the third greatest global health risk after obesity and unsafe sex [1]. Anemia resulting from iron deficiency affects approximately 2 billion people or 34% of the world population [2]. Iron deficiency anemia most severe stage of iron deficiency (defined as a low hemoglobin concentration with iron deficiency) was found in 3% of the adolescent females in the United State of America [3].

Iron deficiency has both physiologic and pathologic causes. Physiologic causes relate to the greater iron demands during periods of growth and development whereas pathologic causes refer to iron losses secondary to a chronic medical condition. In general, iron deficiency results when iron demands by the body are not met by iron absorption. Thus, iron deficiency can result from inadequate intake, impaired absorption, increased requirements, and chronic blood loss.

More than half of the world's undernourished population lives in India [4] and half of Indian children and women are malnourished [5]. Apart from overall poverty and lower literacy rate the health status of women in India reflects gender discrimination from birth [6]. Intrafamilial food distribution, where the males are privileged with high quality nutritious food and the females are deprived of it, is seen in India. Moreover, early and frequent reproductive cycling and presence of reproductive tract infections in adolescent girls lead to iron deficiency anemia [7]. Among females, menstrual blood loss becomes an issue and heavy loss (>80 mL/month) is a significant risk factor. Menstrual blood loss averages about 20 mg of iron per month and in some individuals be as high as 58 mg [8]. In spite of increased iron needs many adolescent girls have iron intake of only 10-11 mg/day, resulting in approximately 1 mg of absorption of iron. About three fourth of adolescent females do not meet the dietary requirements [8].

Iron deficiency is a systemic condition which has many non ematological consequences, which occurs in relation to its severity, like decreased physical work capacity [9], decreased athletic performance [10], lowered indurations [11], depressed immune function [12], decreased scholastic performance, compromised growth and development, and increased risk of pregnancy complication including prematurity and total growth retardation and impaired cognitive function [13–15].

In present study, effort has been made to assess the effect of iron deficiency on cognitive functions in anemic iron deficient and in nonanemic iron deficient school going adolescent girls in a village school.

## 2. Materials and Methods

The present study was carried out in the Department of Pathology, MGIMS, Sevagram, India from July 2007 to September 2009. Approval was obtained from the Institutional Ethics Committee for the study.

### 2.1. Selection of Subjects

A secondary school in the neighborhood, having girl students in the age group of 12−15 years studying in sixth to ninth standard was selected. Necessary permission was taken from the school authority and girls were explained in detail about the study in the school assembly. Participation in the screening programme was voluntary. An explanatory letter and consent form was given to all the girls. Written consent was obtained from parents or guardian for participation in the screening as all participants were minor. Participants completed a questionnaire asking for significant family, medical and menstrual history, parent education, and their dietary habits.

### 2.2. Method of Screening

Screening for anemia and iron deficiency was done by (1) complete blood count: done by automatic cell counter, that is, Coulter for hemoglobin concentration. (2) estimation of serum ferritin concentration was done by ELISA. For this recommended protocol by the kit used was followed. Established age adjusted values for hemoglobin and serum ferritin were used.

The participants after screening were divided into three groups.

1. *Group I*—participants who were anemic (Hb < 12 gm%) and iron deficient (serum ferritin less than 12 *μ*g/L).
2. *Group II*—participants who were nonanemic (Hb ≥ 12 gm%) and iron deficient (serum ferritin less than 12 *μ*g/L).
3. *Group III*—participants who were nonanemic (Hb ≥ 12 gm%) and noniron deficient (serum ferritin levels of 12 *μ*g/L or more).

### 2.3. Method of Assessing the Cognitive Function

After dividing the participants into three groups, that is, anemic iron deficient (group I), nonanemic iron deficient (group II), and nonanemic noniron deficient (group III), for assessing the cognitive function mathematics score, one multicomponent test for memory, attention and verbal learning, and intelligent quotient (IQ) scores of the students was used.

#### 2.3.1. Scholastic Assessment (Mathematics Score)

For assessment of scholastic performance, the mathematics score obtained in the final term examination was noted from the report card. The score obtained were from total 100 marks.

#### 2.3.2. Multicomponent Test (MCT) for Verbal Learning, Memory, and Attention

Multicomponent test of the three groups was assessed after randomization by using PGI test—(Dr. N. N. Wig & Dr. Dwarka Prasad) for testing memory attention and verbal learning [16] Both participants and the investigators were unaware of the group assignment.

*PGI test*—(Dr. N. N. Wig & Dr. Dwarka Prasad), consisted of the following ten subtests: (I) remote memory, (II) recent memory, (III) mental balance, (IV) attention and concentration, (V) delayed recall, (VI) immediate memory, and (VII) verbal retention for similar pairs, (VIII) verbal retention with dissimilar pairs, (IX) visual retention, and (X) recognition.

#### 2.3.3. For Intelligent Quotient (IQ) Assessment

For assessing the intelligent quotient of the girl student's, Bhatia battery performance test—(Dr. C. M. Bhatia) for intelligent quotient (I.Q) was used, which includes two subtest Kohl's block design and Pass along test [17].

All these tests were selected because these have Indian norms and are constructed and standardized in India. *Statistical analysis*: the data was analyzed with SPSS (version 16) statistical software. One-way ANOVAs test (Table 2) and test of significance for comparison of two sample means (Tables 3 and 4) were applied; *P* value and mean and standard deviation were calculated.

### 2.4. Results

Out of total 110 girl students in the age group of 12–15 years, consent was obtained from 103 students to participate in the study. Subsequently 100 students were tested for hemoglobin concentration, serum ferritin, and cognitive function. (3 students refused to give blood for test.)

63 of 100 girl students had hemoglobin levels less than 12 gm% and 37 had hemoglobin levels above 12 gm%. Thus, prevalence of anemia in school going adolescent girls was 63%. The overall mean hemoglobin in the study was 11.66 ± 1.27 g/dL.

Out of the 63 girl students who had anemia, 56 girls (56%) had Hb values between 10 and 12 gm%, 5 girls (5%) had Hb values between 7 and 10 gm%, and rest of the 2 girls (2%) had Hb values below 7 gm%. Thus, mild anemia (Hb 10–12 gm%) was present in 56% of the study subjects, moderate anemia (Hb 7–10 gm%) in 5%, and severe anemia (Hb < 7 gm%) was present in only 2% of the study subjects.

Serum ferritin was done in 100 girl students between the age group of 12−15 years, 67 were iron deficient (serum ferritin <12 *μ*g/L). So the prevalence of iron deficiency was 67%.

Out of 63 girls who were anemic, 50 girls had reduced serum ferritin. Since the total number of students was 100 thus, the prevalence of iron deficiency anemia was 50% and rest of the 13 girls who were anemic, the cause of anemia was other than iron deficiency (see Table 1). To know the probable cause of anemia in these 13 girls who were anemic but were not iron deficient, further investigation in the form of peripheral smear examination, reticulocyte count, and electrophoresis was done. Sickle cell trait was found in 4 out of the 13 students, 1 was sickle cell disease, 1 was thalassemia minor, and 7 were macrocytic anemia probably due to vitamin B12 and folic acid deficiency; hence, they were excluded from the study.

For assessing the cognitive function, mathematics score, one multicomponent test for verbal learning, attention and memory, and IQ scores was used. For assessing the scholastic performance, the mathematics scores obtained in the final term examination was noted from the report card. The scores obtained were from a total of 100 marks. Mean mathematic score calculated for three groups.

The difference in mathematics score was highly significant (*P* value < 0.0001) between nonanemic noniron deficient (group III) and anemic iron deficient (group I), it was significant (*P* value 0.001) between nonanemic noniron deficient (group III) and nonanemic iron deficient (group II).

Multicomponent test (MCT) of verbal learning, attention, memory and IQ scores of all the three groups was assessed after randomization. Tests were administered under the guidance of trained research assistant of the Department of Psychological Medicine. The person who was assessing the tests was unaware of the group assignment.

The difference in scores of mental balance between the nonanemic noniron deficient (group III) and anemic iron deficient (group I) was significant (*P* value < 0.0001) and also the scores of mental balance differed significantly (*P* value 0.002) between the nonanemic noniron deficient (group III) and nonanemic iron deficient (group II). The difference in scores of attention and concentration was significant (*P* value < 0.0001) between non anemic noniron deficient (group III) and anemic iron deficient (group I). And the nonanemic iron deficient (group II) and nonanemic noniron deficient (group III) also showed statistically significant (*P* value 0.004) difference in scores of attention and concentration.

Non anemic non iron deficient (group III) and anemic iron deficient (group I) when compared for scores of Verbal Retention for Similar Pairs showed significant difference (*P* value < 0.0001), and on comparing the scores of Verbal Retention for Similar Pairs between the non-anemic iron deficient (group II) and non anemic non iron deficient (group III) the difference was also statistically significant (*P* value 0.004).

The difference in scores of verbal retention for dissimilar pairs between the anemic iron deficient (group I) and nonanemic iron deficient (group III) was statistically significant (*P* value 0.004) and also between the nonanemic iron deficient (group II) and nonanemic noniron deficient (group III) showed statistically significant difference (*P* value 0.045).

The scores of recognition between the anemic iron deficient (group I) and nonanemic iron deficient (group III) showed significant difference (*P* value 0.032), similarly the difference in scores of recognition between the nonanemic iron deficient (group II) and nonanemic iron deficient (group III) were statistically significant (*P* value 0.04).

For assessing the intelligent quotient (IQ) of the girl students two test, that is, Kohl's block design test and Pass along test were used. After obtaining the test quotient (TQ) from these two tests the IQ was calculated.

The IQ levels differed significantly between nonanemic noniron deficient (group III) and anemic iron deficient (group I) with *P* value < 0.0001 and also between nonanemic noniron deficient (group III) and nonanemic iron deficient (group II) (*P* value 0.003).

Thus, the cognitive function scores which included the mathematics score, multicomponent test scores, and IQ scores were less in iron deficient both anemic and nonanemic groups (group I and II) than the noniron deficient nonanemic group (group III).

## 3. Discussion

Although all the features of cognition are important but verbal learning and attention and concentration along with memory are particularly more important for academic performance.

### 3.1. Scholastic Assessment (Mathematics Scores)

In present study, iron deficient both anemic and nonanemic students had scored less in mathematics than the normal non iron deficient students. This is in accordance to the study done by Prestonjee [18] wherein iron deficient adolescents both anemic and non anemic had lower mathematics score compared with adolescent with normal iron status. Similarly Sungthong et al. [19] observed that the school performance including Thai language and mathematics score were less in iron deficient children than in non iron deficient children in a study done on school children in Thailand.

### 3.2. Multicomponent Test for Verbal Learning, Memory, and Attention

Multicomponent test of verbal learning, attention, and memory of all the three groups was assessed after randomization. The overall total score was less in iron deficient both anemic and nonanemic groups than noniron deficient nonanemic group.

Similar findings were also seen in scores of mental balance and verbal retention for similar and dissimilar pairs. There was no difference in score of recent and remote memory, delayed and immediate recall, and visual retention subsets between iron deficient (group I and II) and noniron deficient (group III).

The findings suggest that iron deficiency, even in the absence of anemia causes decrease in at least some aspect of cognitive functioning.

The present findings are in accordance with the findings of a randomized trial done by Bruner et al. [20] on nonanemic iron deficient adolescent girls in four Baltimore high schools in USA, where baseline cognitive function was assessed. The investigators then randomly assigned these girls to either placebo or oral ferrous sulphate treatment for 8 weeks. The girls treated with ferrous sulphate had improved scores in verbal learning and memory compared to the scores of girls who were given placebo. Similar observation was also seen in the study done by Seshadri et al. [21] in India who showed beneficial effect of iron therapy on cognitive function of anemic children of various age. In anemic adolescent girls of 8–15 years of age group on iron therapy there was improvement in the scores of attention, memory, and concentration than those girls who were given placebo. A study done in Vadodara, India by Sen, and Kanani present evidence from a controlled intervention trial that iron and folic acid supplementation in children aged between 9 and 13 years leads to modest (1.5 to 2 units on a scale of 100) but significant improvement in the various cognitive tests [22].

### 3.3. Intelligent Quotient Assessment

The difference of mean IQ scores between iron deficient both anemic and nonanemic groups (I and II) and nonanemic noniron deficient was statistically significant.

Pollitt et al. [23], Soemantri et al. [24], and Sunthong et al. [19] also reported low IQ scores in iron deficient subjects compared to the noniron deficient subjects. This is in accordance with present study findings.

## 4. Conclusion

The findings of the present study are iron deficient school going adolescent females both anemic and nonanemic had low scholastic performance in the form of low mathematics score and low scores in verbal learning, attention, mental balance, and recognition component of multicomponent test along with low IQ scores than their noniron deficient comparers. Iron deficiency independently leads to decreased cognitive scores. Iron deficiency without anemia is the initial stage and as the iron deficiency increases anemia manifests. The cognitive scores were lowest in the iron deficient anemic (group I). In iron deficient without anemia it was slightly more than group I but much less than group III, that is, nonanemic noniron deficient subjects. Prevalence of anemia was 63% while prevalence of iron deficiency anemia in present study was 50%.

There is need to initiate programme for supplementation of iron and folic acid to school going adolescent girls for the prevention of hematological and nonhematological consequences of iron deficiency with government and private organizational efforts.

## Figures and Tables

**Table 1:** Number of students in different groups, mean serum ferritin, and hemoglobin levels.

Groups	Number of students	Mean serum ferritin (*µ*g/L)	Mean hemoglobin levels (gm%)
Anemic iron deficient (group I)	50	8.458	11.01
Nonanemic iron deficient (group II)	17	10.02	12.61
Nonanemic noniron deficient (group III)	20	25.275	12.975

**Table 2:** The mean mathematics score of the girl students in different groups.

Groups	Mean mathematics score ± SD
Anemic iron deficient (group I) (*n* = 50)	47.76 ± 8.26
Nonanemic iron deficient (group II) (*n* = 17)	52.64 ± 9.88
Nonanemic noniron deficient (group III) (*n* = 20)	62.15 ± 5.93

SD: Standard deviation.

**Table 3:** The mean multicomponent test (MCT) scores in the three different groups.

Test	Anemic iron deficient (group I) *N* = 50 (Mean ± SD)	Nonanemic iron deficient (group II) *N* = 17 (Mean ± SD)	Nonanemic noniron deficient (group III) *N* = 20 (Mean ± SD)
Recent memory	4.98 ± 0.14	5 ± 0	4.95 ± 0.22
Remote memory	5.82 ± 0.52	5.88 ± 0.48	5.6 ± 0.75
Mental balance	1.66 ± 0.55	2.05 ± 0.74	2.7 ± 0.47
Attention and concentration	11.28 ± 3.83	13.76 ± 3.41	16.2 ± 2.06
Delayed recall	8.28 ± 1.48	8.48 ± 1.85	8.9 ± 1.07
Immediate recall	1.78 ± 0.73	1.82 ± 0.72	1.9 ± 0.78
Verbal retention for similar pairs	4.1 ± 0.64	4.23 ± 0.83	4.9 ± 0.30
Verbal retention for dissimilar pairs	4.3 ± 0.67	4.35 ± 0.49	4.8 ± 0.41
Visual retention	2.36 ± 1.13	2.41 ± 1.46	2.85 ± 1.34
Recognition	7.7 ± 1.84	7.87 ± 1.66	8.95 ± 2.39
Total test scores	52.26 ± 5.73	55.82 ± 6.12	61.75 ± 4.92

SD: Standard deviation.

**Table 4:** The mean scores in Kohl's block design, pass along test, and mean IQ score.

Groups	Kohl's block design	Pass along test	Test quotient (TQ)	Mean intelligent quotient (±SD)
Anemic iron deficient (group I)	11.86	12.76	193.06	96.6 (±7.28)
Nonanemic iron deficient (group II)	12.82	15.47	205.17	102.61 (±6.33)
Nonanemic noniron deficient (group III)	14.4	16.9	215.65	107.82 (±4.95)

SD: Standard deviation.
